# Public awareness should be raised on a crucial but neglected factor for COVID-19 vaccination

**DOI:** 10.3389/fimmu.2022.1027539

**Published:** 2022-09-26

**Authors:** Hao Yuan, Yining Song, Xiu-Xiang Zhang, Jingbo Zhai, Jin Zhang, Zi-Guo Yuan

**Affiliations:** ^1^ Guangdong Provincial Key Laboratory of Zoonosis Prevention and Control, College of Veterinary Medicine, South China Agricultural University, Guangzhou, China; ^2^ Medical College, Inner Mongolia Minzu University, Tongliao, China; ^3^ Key Laboratory of Zoonose Prevention and Control at Universities of Inner Mongolia Autonomous Region, Tongliao, China; ^4^ Department of Pharmacy and Toxicology, University of Missippi Medical Center, Jackson, MS, United States

**Keywords:** COVID19, vaccination, neglected factor, public awareness, Toxoplasma gondii, immunosuppressive pathogens

According to the current epidemic trend, herd immunity can be achieved *via* a vaccination program on a wide scale, representing one of the important ways to block the spread of COVID-19 (Corona Virus Disease 2019). Herd immunity is largely affected by the frequency of vaccination and the type of vaccine. Currently, the low vaccine protection rate is mostly attributed to a) there is no vaccine for children under 6 months, and exemption of partial population with neurologic disorder and anaphylactic disease and immunocompromised patients from receiving the vaccine, and b) the emergence of variant strains across the world has greatly reduced the protection of the vaccine. In addition, a significant factor may be neglected: the influence of immunosuppressive parasite infection.

As of August 2022. 343.0624 million COVID-19 vaccines have been vaccinated across China (1). Due to the differences in the coverage of COVID-19 vaccines worldwide and the prevalence of delta mutants and Omicron. Due to decreased vaccine protective efficacy in humans over time, new cases still emerge in an endless stream. In the interim analysis data of Phase III clinical trial released by Johnson & Johnson Ad26 adenovirus vector COVID-19 vaccine on January 29, 2021, 468 of the 43,783 subjects were infected with COVID-19. This unique pattern raises an important question, why does infection still occur during the period of antibody protection after vaccination? a) It may be related to antibody production time and titer. b) immunosuppressive parasite infection could be an important factor that has been ignored and never been investigated as a potential cause of vaccine failure.

Nowadays, with the development of the economy, people are petting cats for emotional support (2). Feline is the definitive host of *Toxoplasma gondii* (*T. gondii*), where oocysts, the infective stage, can be discharged, causing environmental contamination and widespread infection. According to statistics, the seroprevalence of *T. gondii* varies from less than 10-60% in the world’s nations (3). *T. gondii*, as a zoonotic parasitic disease, acute or chronic infections, can cause systemic or local immunosuppression of the host. Studies have shown that 65% of patients with HIV die from the re-activation of *T. gondii* infection in the first year after diagnosis (4). It is well documented that *T. gondii* has developed mechanisms to evade the attack by the host immune system (5, 6). Serological testing for *T. gondii* is not compulsory, and there is no *T. gondii* vaccine. In the case of such a high seropositivity rate, the antibody titers of the people injected with COVID-19 vaccines may be affected by suppressive *T. gondii* infection. We hypothesize that this could be one of the important neglected reasons for the low vaccine protection rate and should raise the attention of the Centers for Disease Control and Prevention worldwide.

Even though we are unsure whether *T. gondii* infection is connected to COVID-19 vaccination failure, we should test for immunosuppressive pathogens like *T. gondii* in vaccination failure patients to confirm the connection between vaccination failure and immunosuppression and to increase the efficacy and protection rate of COVID-19 vaccines.

## Author contributions

HY, YS, X-XZ, and JZ are responsible for writing the draft. JZ is responsible for revising the manuscript. Z-GY is responsible for the conception and polishing the MS. All authors contributed to the article and approved the submitted version.

## Funding

This work was supported by the National Natural Science Foundation of China (31972707) and Guangdong Provincial Forestry Department's Provincial Financial Special Fund for Ecological Forestry Construction-Wildlife Conservation.

## Acknowledgments

We sincerely thank the support of Yasser Mahmmod for his overview and precious suggestion on the article.

## Conflict of interest

The authors declare that the research was conducted in the absence of any commercial or financial relationships that could be construed as a potential conflict of interest.

## Publisher’s note

All claims expressed in this article are solely those of the authors and do not necessarily represent those of their affiliated organizations, or those of the publisher, the editors and the reviewers. Any product that may be evaluated in this article, or claim that may be made by its manufacturer, is not guaranteed or endorsed by the publisher.
